# In Vitro Genotoxicity Evaluation of an Antiseptic Formulation Containing Kaolin and Silver Nanoparticles

**DOI:** 10.3390/nano12060914

**Published:** 2022-03-10

**Authors:** Adriana Rodriguez-Garraus, Amaya Azqueta, Francisco Laborda, Ana C. Gimenez-Ingalaturre, Alba Ezquerra, Luis Lostao, Adela Lopez de Cerain

**Affiliations:** 1 Department of Pharmacology and Toxicology, School of Pharmacy and Nutrition, University of Navarra, Irunlarrea 1, 31008 Pamplona, Spain; arodriguez.53@alumni.unav.es (A.R.-G.); acerain@unav.es (A.L.d.C.); 2 Navarra Institute for Health Research, IdiSNA, Irunlarrea 3, 31008 Pamplona, Spain; 3 Group of Analytical Spectroscopy and Sensors (GEAS), Institute of Environmental Sciences (IUCA), University of Zaragoza, Pedro Cerbuna 12, 50009 Zaragoza, Spain; flaborda@unizar.es (F.L.); acgimenez@posta.unizar.es (A.C.G.-I.); 4 Enosan Laboratories, Maria de Luna 11, 50018 Zaragoza, Spain; investigacion@laboratoriosenosan.com (A.E.); llostaoca@gmail.com (L.L.)

**Keywords:** silver nanoparticles, kaolin, genotoxicity

## Abstract

Worldwide antimicrobial resistance is partly caused by the overuse of antibiotics as growth promoters. Based on the known bactericidal effect of silver, a new material containing silver in a clay base was developed to be used as feed additive. An in vitro genotoxicity evaluation of this silver-kaolin clay formulation was conducted, which included the mouse lymphoma assay in L5178Y TK^+/−^ cells and the micronucleus test in TK6 cells, following the principles of the OECD guidelines 490 and 487, respectively. As a complement, the standard and Fpg-modified comet assays for the evaluation of strand breaks, alkali labile sites and oxidative DNA damage were also performed in TK6 cells. The formulation was tested without metabolic activation after an exposure of 3 h and 24 h; its corresponding release in medium, after the continuous agitation of the silver-kaolin for 24 h was also evaluated. Under the conditions tested, the test compound did not produce gene mutations, chromosomal aberrations or DNA damage (i.e., strand breaks, alkali labile sites or oxidized bases). Considering the results obtained in the present study, the formulation seems to be a promising material to be used as antimicrobial in animal feed.

## 1. Introduction

Antimicrobial resistance is an economic and security threat which will reach worrying dimensions by 2050 unless solutions are found [1,2,3]. Multidrug-resistant pathogens began to originate worldwide in the 1980s [4]. One of the main reasons of antimicrobial resistance development is the therapeutic and non-therapeutic overuse of antimicrobials in humans, agriculture, and companion and food animals [1,2,5]. More specifically, the use of long-term and low-dose antimicrobials in animal production as growth promoters is an important contributor to antimicrobial resistance occurrence and its spread between animals, humans, and the environment [5,6,7,8].

In 1999, the European commission highlighted the need of facing antimicrobials use as growth promoters. Later on, in 2006, their banishment entered into force without alternatives [9,10,11]. Silver nanoparticles (AgNPs) are turning out to be a good alternative to antibiotics and their use has increased in the food sector [12,13,14,15]. AgNPs have demonstrated to exert bactericidal activity against both Gram-positive and Gram-negative antibiotic-resistant bacteria [16,17,18,19,20,21,22,23]. Moreover, it has been demonstrated that AgNPs are effective against species that cause infectious diseases in poultry, such as *Bacillus subtilis*, *Escherichia coli*, *Salmonella typhimurium*, *Staphylococcus aureus*, and *Pseudomonas aeruginosa* [23]. Furthermore, when low doses of AgNPs were added to feed for pigs, a linear daily growth increase of the animals was observed, together with a reduction in the population of ileal coliform bacteria [24]. Based on the known bactericidal effect of silver, a new material was developed to be used as a feed additive, which was composed of kaolin as support for AgNPs, (Feed additive for animals: Spanish National patent N° 200701496 and Nanosystems comprising silver and antibiotics and their use for the treatment of bacterial infections: Patent in international extension China, Japan, USA, EPO, Brazil, Colombia and Mexico, N° PCT/EP2018/059006). This silver-kaolin formulation exhibited activity against a wide spectrum of Gram-negative and Gram-positive bacteria, including multidrug-resistant strains [25]. The use of the silver-kaolin formulation as alternative to antibiotics in livestock farms would contribute to a decrease in antimicrobial resistance problem caused by antibiotics overuse in animals.

Other materials similar to the silver-kaolin formulation of the present study have also shown considerable antimicrobial effects. AgNPs immobilized onto sodic montmorillonite clay showed antimicrobial effect against *Escherichia coli* and *Staphylococcus aureus* [26]. Also, silica-silver core-shell nanoparticles exerted inhibitory effects on *Escherichia coli* and *Bacillus subtilis*, and kaolin clay loaded with AgNPs was highly effective against *Escherichia coli* and *Salmonella* ssp. [27,28]. Moreover, after feeding broiler chicks with nano-silica-silver at 2, 4, or 8 mg/kg for 35 days, Dosoki and colleagues suggested that this material could be used as a dietary supplement at the dose of 4 mg/kg due to its anti-inflammatory, antimicrobial, and immuno-stimulatory properties [29]. Furthermore, the survival rate of Salmonella-infected chicks fed with AgNPs was 10%, and below this value in those that did not receive any treatment. In contrast, the addition of nano-silica platelets resulted in a dramatic increase in the survival rate, which was up to 50% and even 75% in those animals fed with nano-silica platelets and AgNPs-nano-silica platelets, respectively [30].

Although the bactericidal activity of similar materials has been demonstrated, the safety of all these materials must also be ensured. Therefore, the introduction of novel materials into the food sector such as, nanomaterials (NMs), requires their safety evaluation, as well as a clearer understanding of any potential impact on animal or human health. In 2018, the European Food Safety Authority (EFSA) published a guidance on risk assessment of the application of nanoscience and nanotechnologies in the food and feed chain [31]. Within the risk and safety assessment of a compound, genotoxicity evaluation is essential to identify compounds that induce DNA lesions, gene mutations, and chromosomal aberrations.

AgNPs genotoxicity studies were recently reviewed by Rodriguez-Garraus and colleagues, and AgNPs’ ability to produce gene mutations, chromosome aberrations, and DNA damage was evidenced [32]. In this regard, the security of any material containing AgNPs needs to be thoroughly assessed.

On the other hand, kaolin is considered as an inert material and in consequence, studies about its potential genotoxicity are very scarce. Li and colleagues investigated the safety of a material based on nano-silica platelets by the in vitro comet assay, the in vivo micronucleus (MN) test and the Ames test [33]. In this study, CHO cells were treated for 24 h with 62.5–1000 µg/mL nano-silica platelets obtaining negative results in the three genotoxicity assays. Surprisingly, positive in vitro genotoxic responses were observed in MN test when evaluating several kaolin materials i.e., micro and nano-kaolin particles at 0.2–200 µg/mL after 6 h treatment in A549 cells; or in CHO AA8, HEKn, and FJ cells [34,35]. Pseudohexagonal and spherical micro-kaolin material at 100 µg/mL, after 1 h treatment in A549 and A549-RAW764 co-culture, also led to positive results in standard and Fpg-modified comet assay [36].

Safety determinations of clays combined with AgNPs are scarce and genotoxicity studies of materials composed of both AgNPs and kaolin have not been found. In this regard, the genotoxicity evaluation of AgNPs-kaolin composites intended for the feed industry is of great interest. Given the evidence of in vitro genotoxic effects of AgNPs and the promising application of kaolin as an inert support, the aim of this study is to demonstrate the reduction of genotoxicity of AgNPs when they are formulated as a combinationwith kaolin. To that end, the in vitro genotoxicity study of silver-kaolin formulation was carried out, following the EFSA 2018 guideline testing strategy, and the principles of the corresponding OECD guidelines. The evaluation was complemented by the also recommended comet assay for the evaluation of nanomaterials known to produce DNA damage by oxidation.

## 2. Materials and Methods

All assays were carried out in Good Laboratory Practices (GLP)-accredited laboratory, and all methods were carried out under GLP-like conditions.

### 2.1. Chemicals and Reagents

Silver-kaolin formulation (Laboratorios ENOSAN, Zaragoza, Spain). Formamide pyrimidine DNA-glycosylase (Fpg) (NorGenoTech, Oslo, Norway). RPMI-1640 medium containing D-glucose, HEPES, L-glutamine, sodium bicarbonate and sodium pyruvate (ref. A10491-01), phosphate buffer saline (PBS) and Pluronic F-68 10% (Gibco-Thermo Fisher Scientific, Madrid, Spain). Colchicine, Ethidium Monoazide Bromide (EMA) dye, sytox-green dye, RNAase and beads (Invitrogen-Thermo Fisher Scientific, Waltham, MA, USA). Penicillin-streptomycin (Lonza, Basel, Switzerland). Heat-inactivated horse serum (HIHS), hypoxanthine, glycine, methotrexate, thymidine, 5-trifluorothymidine (TFT), methyl methane sulfonate (MMS), fetal bovine serum (FBS), NaCl, trisodium citrate dihydrate, NaOH, KCl, IGEPAL, sucrose, dimethyl sulfoxide (DMSO), citric acid, low melting point agarose (LMP), standard agarose, Triton X-100, Tris base, HEPES, Na_2_EDTA, bovine serum albumin (BSA) and 4,6-diamidino-2-phenylindole (DAPI) (Sigma-Aldrich, Darmstadt, Germany). Potassium bromate (KBrO_3_) (Merck, Darmstadt, Germany).

### 2.2. Characterization of the Silver-Kaolin Formulation

Commercial kaolin was treated through a method under patent (ENOSAN), which enables silver (Ag) to be deposited on its surface as AgNPs. The resulting material is a kaolin formulation containing AgNPs attached to its surface.

The crystalline phase composition of the silver-kaolin formulation was determined by X-ray diffraction (XRD), its granulometry by X-ray absorption sedimentation, and the stability of aqueous suspensions by dynamic light scattering (DLS). The presence, morphology, and size distribution of AgNPs were studied by field emission scanning electron microscopy (FESEM). The Ag content in the formulation and its release in aqueous media were determined by flame atomic absorption spectrometry (F-AAS). The fractionation of the released silver as dissolved, and also as particulate was carried out by single particle inductively coupled plasma mass spectrometry (SP-ICP-MS).

### 2.3. Test Compound Preparation

The test compound was studied at different concentrations in two different forms: the silver-kaolin formulation directly added to the cell cultures, and the release after the continuous agitation of the silver-kaolin formulation in the corresponding cell treatment medium for 24 h, called silver-kaolin release. To this aim, silver-kaolin formulation at the corresponding concentration was added to flasks containing culture medium and maintained in a shaking incubator at 37 °C, 5% CO_2_ for 24 h. Then, flask content was centrifuged (411× *g*, 5 min), and the supernatant was recovered to be immediately used for cell treatment.

### 2.4. Cell Lines and Cell Culture

The mouse lymphoma assay (MLA) was conducted using mouse lymphoma L5178Y TK^+/−^ cells, obtained from the American Type Culture Collection [L5178Y TK+/(clone3.7.2C)] (ATCC^®^ CRL9518™)). Growth medium was RPMI-1640 medium supplemented with 100 U/mL penicillin, 100 µg/mL streptomycin, 0.1% Pluronic F-68, and 10% HIHS (ML10). Medium with the same composition but supplemented with 5% or 20% of HIHS (ML5 and ML20, respectively) were also prepared to perform the assay.

The MN test and the comet assay were performed in TK6 cells obtained from the ATCC (ATCC^®^ CRL8015™). TK6 growth media was composed by RPMI-1640 medium supplemented with 100 U/mL penicillin, 100 µg/mL streptomycin, and 10% FBS.

Cells were maintained in a humidified atmosphere, using gentle shaking at 37 °C and 5% CO_2_ at a density of 2 × 10^5^–1 × 10^6^ cells/mL and subcultured for a maximum of two months; then, if needed, new vials were defrosted.

### 2.5. Cytotoxicity

Cytotoxicity of silver-kaolin formulation was evaluated using the proliferation assay in L5178Y TK^+/−^ and TK6 cells. Each proliferation assay consisted in a negative control (i.e., untreated cells) and 5 concentrations of silver-kaolin formulation. Three independent experiments were performed.

The silver-kaolin formulation concentrations tested in L5178Y TK^+/−^ cells were 0.12, 0.37, 1.11, 3.33, and 10 mg/mL for 3 h and 24 h. The ones tested in TK6 cells were 0.31, 0.63, 1.25, 2.5, and 5 mg/mL for 3 h and 0.01, 0.02, 0.06, 0.17, and 0.5 mg/mL for 24 h. After each treatment, L5178Y TK^+/−^ cells were counted and their survival % was calculated in comparison to the negative control. Then, L5178Y TK^+/−^ cells were washed by 5 min decantation and adjusted to 2 × 10^5^ cells/mL in ML20. After 24 h incubation, cells were readjusted to 2 × 10^5^ cells/mL in ML20. At last, cells were counted after another 24 h incubation. Regarding TK6 cells, they were centrifuged after treatment (5 min, 293× *g*) seeded at 2 × 10^5^ cells/mL in fresh medium and let grow for 24 h. Afterwards, cells were seeded again at 2 × 10^5^ cells/mL and placed in the incubator for another 24 h, when they were finally counted. As a measure of viability, total suspension growth (*TSG*) and the relative suspension growth (*RSG* %) were calculated for each assay condition, according to the following formulae.

$$TSG=\frac{total\;no.\;cells\;48\;h\;after\;treatment}{total\;no.\;cells\;before\;treatment}$$

$$RSG\;\%=\frac{TSG_{treated}}{TSG_{control}}\times 100$$

Result of the proliferation assays with silver-kaolin formulation permitted the choice of the concentration ranges exerting acceptable levels of cytotoxicity for each cell line. Then, proliferation assays were carried out testing silver-kaolin release, see Section 2.3). The silver-kaolin formulation concentrations used for silver-kaolin release and tested in L5178Y TK^+/−^ cells were 0.12, 0.37, 1.11, 3.33, and 10 mg/mL for 3 h and 0.03, 0.07, 0.22, 0.67, and 2 mg/mL for 24 h treatment. The silver-kaolin formulation concentrations used for silver-kaolin release and tested in TK6 cells were 0.02, 0.06, 0.17, 0.5, and 1.5 mg/mL for 3 h and 0.01, 0.02, 0.06, 0.17, and 0.5 mg/mL for 24 h.

### 2.6. Mouse Lymphoma Assay

The MLA was carried out following the principles of the OCED TG 490 in its microwell version [37].

#### 2.6.1. Mutant L5178Y TK^−/−^ Cleansing

As a preliminary step, to maintain the mutation frequency (MF) within the 50–170 × 10^−6^ range required by the OECD TG 490, L5178Y TK^+/−^ cells were cleansed to eliminate Tk^−/−^ mutants and therefore increase the proportion of Tk^+/−^ cells [37,38,39]. A total of 1 × 10^7^ cells were grown for 24 h in THGM medium (a variation of ML10, containing 9 µg/mL thymidine, 15 µg/mL hypoxanthine, 22.5 µg/mL glycine and 0.4 µg/mL methotrexate). Then, cells were centrifuged, washed with RPMI medium, resuspended in THG medium (a variation of ML10, containing 9 µg/mL thymidine, 15 µg/mL hypoxanthine, 22.5 µg/mL glycine) at a concentration of 1 × 10^5^ cells/mL, and incubated for 48 h. In between, cells were counted after 24 h, seeded again to a density of 2 × 10^5^ cells/mL in THG medium. Finally, 48 h after the end of the treatment, MF was determined by MLA (see Section 2.6.2 and Section 2.6.3) and a stock of cleansed cells was frozen at −140 °C in aliquots of 1 mL at a concentration of 6 × 10^6^ cells/mL in a ML10 containing 5% DMSO.

#### 2.6.2. Mouse Lymphoma Assay

Each experiment consisted of one negative control (untreated cells), one positive control (cells treated with 100 µM MMS), and cells treated with 4 concentrations of the silver-kaolin formulation or with the corresponding silver-kaolin release (see Section 2.3). The following concentrations were assayed: 0.12, 0.37, 1.11, and 3.33 mg/mL for the short treatment and 0.07, 0.22, 0.67, and 2 mg/mL for the long one.

L5178Y TK^+/−^ cells were treated for 3 h and 24 h (short treatment in ML5 and long treatment in ML10, respectively). For each test condition, 1 × 10^7^ cells were exposed to the silver-kaolin formulation or to the corresponding silver-kaolin release. For the short treatment, those cells were treated at a concentration of 1 × 10^6^ cells/mL, in T25 flasks. For the long treatment, the 1 × 10^7^ cells were exposed to the different treatment conditions at a density of 5 × 10^5^ cells/mL, in T75 flasks. After treatment, cells were washed by 5 min decantation. Positive control cells were washed twice with PBS by centrifugation (5 min, 293× *g*, 4 °C) and seeded in fresh medium.

After washing, cells were diluted to 2 × 10^5^ cells/mL in 10 mL of ML20 and they were maintained in T25 flasks at 37 °C and 5% CO_2_ in a humidified atmosphere, by gently shaking. After 24 h, cells were diluted again to 2 × 10^5^ cells/mL. After another 24 h, two different cell suspensions were prepared per cell culture: one at a density of 10,000 cells/mL to score mutant cells, and the second one at a density of 100 cells/mL, to score viable cells. The cell suspensions destined to mutant cells scoring were exposed to 400 µg/mL TFT, the selective agent. Both cell suspensions were seeded in 96-well plates: 2000 cells/well in the mutant plates and 2 cells/well in the viability plates. Two identical plates were seeded per condition.

All the plates were then incubated at 37 °C and 5% CO_2_, in a humidified atmosphere, for 10–12 days, until the colonies were formed. Mutated cell colonies (i.e., TFT resistant cells and so Tk^−/−^ mutants) were visually counted in the mutant plates and cell colonies were counted in the viability plates. Small and large colonies were counted separately. Small colonies were those covering less than 25% of the diameter of the well and large colonies were those covering more than 25% of the diameter of the well [37].

#### 2.6.3. Mouse Lymphoma Assay Calculations

Calculations were made according to the OECD TG 490 [37]. First, cloning efficiency (*CE*) was calculated for the mutant and the viability plates as follows.

$$CE=\frac{\left(-\;ln\;\left(\frac{empty\;wells}{total\;seeded\;wells}\right)\right)\times total\;seeded\;wells}{seeded\;cells\;per\;well}$$

Then, to evaluate mutagenicity, the *MF* was calculated for each assay condition by the application of the following formula, in which “*m*” represents the mutant plates and “*v*” the viability ones.

$$MF=\frac{CEm}{CEv}$$

The relative cloning efficiency (*RCE*) was calculated for each assay condition by the application of the following formula, in which “*t*” stands for treatment and “*c*” for control.

$$RCE=\frac{CEv_{t}}{CEv_{c}}$$

Finally, the relative total growth (*RTG*) was calculated for each assay condition in order to assess cytotoxicity.

$${RTG}_{treatment}=\frac{{RCE}_{t}\times {RSG}_{t}}{100}$$

In all cases, the mean of the two plates of each condition was obtained.

The acceptability of each MLA was evaluated applying the OECD TG 490 recommendations [37]. Regarding the negative control, MF must range between 50 and 170 × 10^−6^, CE must range between 65 and 120%, and TSG must range between 8 and 32-fold for short treatment, and between 32 and 180-fold for the long one. In relation to the positive control, it must show an absolute increase in MF of at least 300 × 10^−6^ above the negative control, and at least 40% of the colonies had to be classified as small. Then, a response is considered to be positive if any of the experimental conditions shows a MF higher than the MF of the negative control plus 126 × 10^−6^, and if the MF increase is concentration-related.

### 2.7. Micronucleus Test

The MN test was carried out following the principles of the OCED TG 487 [40].

Each experiment consisted in a negative control (untreated cells), and 4 concentrations of silver-kaolin formulation (for long treatment) or 4 concentrations of the corresponding silver-kaolin release (for short and long treatment) (see Section 2.3). Each experiment also included a positive control (i.e., cells treated with 100 µM of the clastogen MMS, and cells treated with 10 ng/mL of the aneugen colchicine) for the short and the long treatment, respectively. The following concentrations of silver-kaolin were assayed: 0.07, 0.22, 0.67, and 2 mg/mL for 3 h and 0.02, 0.06, 0.17, and 0.5 mg/mL for 24 h exposure.

In the short treatment, 6 × 10^5^ cells were exposed to each testing condition, for 3 h in a volume of 1 mL in 12-well plates. Afterwards, they were centrifuged (5 min, 293× *g*) and subcultured until 1.5–2 cell cycles. In the long treatment, 3 × 10^5^ cells were exposed for each testing condition for 1.5–2 cell cycles in a volume of 1 mL in 12-well plates. TK6 cell cycle was previously calculated and 24 h was the time corresponding to 1.5–2 cell cycles.

At the end of the experiment, cells were centrifuged (8 min, 263× *g*, 4 °C), resuspended in 120 µL of EMA nucleic acid staining (0.025 mg/mL EMA in PBS/FBS 2%) and exposed to a light source (60 W light) on ice for 20 min, which was separated 30 cm from the cells. Cells were then washed with 4 mL of PBS with 2% FBS through centrifugation (8 min, 263× *g*, 4 °C). After washing, cells were stained by adding 250 µL of lysis solution 1 (0.2 µM Sytox, 1 mg/mL RNAase, 0.584 mg/mL NaCl, 1 mg/mL trisodium citrate dihydrate, 0.3 µL/mL IGEPAL) and incubated in darkness at room temperature for 1 h. Finally, cells were stained by adding another 250 µL of lysis solution 2 (0.2 µM Sytox, 1.5 µL/mL beads, 85.6 mg/mL sucrose, 15 mg/mL citric acid) and incubated in darkness at room temperature for 30 additional minutes.

Samples were stored for a maximum of 48 h in darkness at 4 °C. Nuclei and MN were analyzed by FACS Canto™ II Six colors (BD, East Rutherford, NJ, USA) by scoring 20,000–25,000 nucleated cells. Micronuclei were gated out following the MicroFlow Instructions Manual from Litron Laboratories (Rochester, NY, USA) by using FlowJo^TM^ V10.2 software (BD, NJ, USA). The frequency of *MN* for each of the samples and controls was referred to 1000 genomic nuclei by applying the following formula.

$$MN/1000\;N=\frac{Number\;of\;MN}{Number\;of\;nucleated\;cells}\times 1000$$

Cytotoxicity was calculated for each testing condition by adding a known number of beads to each sample in the lysis solution 2. For this purpose, the ratio nuclei/beads was assessed for each sample, compared to one of the negative controls (0% cytotoxicity), and showed as relative survival (RS) %. The possible interference of the material with the flow cytometry analysis was also evaluated before carrying out the MN test, showing no interferences.

Statistical analysis was carried out with the Stata 12.0 software (Stata, College Station, TX, USA). The total number of MN obtained for each treatment was compared with the negative control through the Chi squared test. Statistical significance was set at *p* < 0.05. In addition, each assay was subjected to a statistical regression test to check if the response obtained was concentration related. Two independent experiments were carried out.

The acceptability of the MN tests was evaluated applying the OECD TG 487 criteria: negative and positive controls were compatible with the historical control data of the toxicology laboratory and positive controls produced a significant increase in response to negative control [40]. As MN were assessed by flow cytometry, results were considered as positive based on three criteria: (1) a statistically significant increase in the *MN/*1000 *N* compared with the negative control, in at least one of the concentrations tested, (2) a three-fold increase in MN at one or more concentrations over the negative control [41], (3) a concentration-related MN increase over the non-cytotoxic range tested (i.e., RS > 40).

### 2.8. Comet Assay

The comet assay was performed following the procedure previously described by Collins and Azqueta [42], with minor modifications.

Each experiment consisted in a negative control (untreated cells), a positive control (1.25 mM KBrO_3_), and 5 concentrations of silver-kaolin formulation or 5 concentrations of the corresponding silver-kaolin release (see Section 2.3). The following concentrations of silver-kaolin formulations were assayed: 0.02, 0.06, 0.17, 0.5, and 1.5 mg/mL for the 3 h treatment and 0.01, 0.02, 0.05, 0.17 and 0.5 mg/mL for the 24 h one. In the short treatment, a total of 6 × 10^5^ cells were exposed to each testing condition for 3 h in a volume of 1 mL in 12-well plates. In the long treatment, a total of 3 × 10^5^ cells were exposed to each testing condition for 24 h in a volume of 1 mL in 12-well plates. When cells were exposed to KBrO_3_, treatment always lasted 3 h.

After treatment, cells were centrifuged (5 min, 293× *g*, 4 °C), diluted in culture medium to 1 × 10^6^ cells/mL, and mixed with 1% low melting point (LMP) agarose (dissolved in PBS), achieving 0.8% LMP agarose. Two drops of 70 µL of the cell suspension per slide were placed on 1% standard agarose pre-coated and a 20 × 20 mm coverslip was placed on top of each drop. Three identical slides were prepared for each testing condition. Slides were kept immersed for 1 h in lysis solution (2.5 M NaCl, 0.1 M EDTA, 10 mM Tris, 1% Triton X-100, adjusted to pH 10 with NaOH) at 4 °C. Then, two slides per testing condition were washed with buffer F (40 mM HEPES, 0.1 M KCl, 0.5 mM EDTA, 0.2 mg/mL BSA, pH 8) three times (5 min each). Afterwards, 45 µL of buffer F or Fpg enzyme (previously titrated [43]) was added to each gel of their corresponding set of slides, and 22 × 22 mm coverslips were put on top of each gel. Fpg and buffer F slides were incubated in a humidified atmosphere, at 37 °C for 1 h. After that, the coverslips were removed and the slides (including the set of slides kept in lysis solution) were immersed in electrophoresis solution (1 mM EDTA, 0.3 M NaOH, pH > 13) for 40 min at 4 °C. Then, slides were subjected to electrophoresis (1 V/cm) for 20 min, at 4 °C and neutralized by washing them with PBS followed by distilled water (10 min, 4 °C each wash). Finally, each gel was stained with 30 µL of 1 µg/mL DAPI solution and comets were analyzed by a fluorescent microscope (Nikon Eclipse 50 i, Tokyo, Japan) using the image analysis system Comet Assay IV (Perceptive instruments, Bury Saint Edmunds, UK). A total of 100 randomly selected cells were analyzed per slide, 50 cells of each duplicate gel. The DNA damage indicator used was tail DNA intensity (% DNA in tail). The % DNA in tail of the 50 comets analyzed per gel was calculated and then, the mean of both medians of each slide was obtained. The slides which remained immersed in lysis solution were used to assess the strand breaks (SBs) and alkali labile sites (ALS). The difference between the median % DNA in tail of the Fpg-treated slides and the one of the buffer F-treated ones was used to calculate the net Fpg-sensitive sites.

Statistical analysis was carried out with the Stata 12.0 software. Three independent experiments were carried out and the mean and SD of each testing condition were obtained. The % DNA in tail of each testing condition was statistically compared with the negative control through the Kruskal–Wallis test. Statistical significance was set at *p* < 0.05. Minimum Information for Reporting Comet Assay (MIRCA) recommendations were followed in this manuscript [44].

## 3. Results

### 3.1. Characterization of the Silver-Kaolin Formulation

The silver-kaolin formulation showed a silver content, determined by F-AAS after acid digestion, of 0.83 ± 0.04% (m/m), with a crystal phase composition of kaolinite (68%), quartz (12%), illite (13%), potassic feldspar (6%), and metallic silver (0.8%) determined by XRD. Micrographs obtained by FESEM (Figure S1 in Supplementary Information) showed the laminar structure of kaolinite microparticles decorated with spheroidal silver nanoparticles with diameters ranging from 2 to 90 nm (average diameter: 27 nm). Less than 2% (m/m) of silver-kaolin microparticles were larger than 25 µm diameter, with ca. 30% below 1 µm (Figure S2 in Supplementary Information). Aqueous dispersions of the silver-kaolin material measured by DLS showed an overtime sedimentation process leaving no particles above 1 µm in suspension. Mass distribution obtained by SC-ICP-MS analysis showed the silver content (internalized or adsorbed by the cells) corresponding to individual cells, in units of attograms/cell (Figure S3 in Supplementary Information). The technique cannot distinguish between dissolved silver and silver nanoparticles inside the cells.

### 3.2. Cytotoxicity

**Figure 1:**
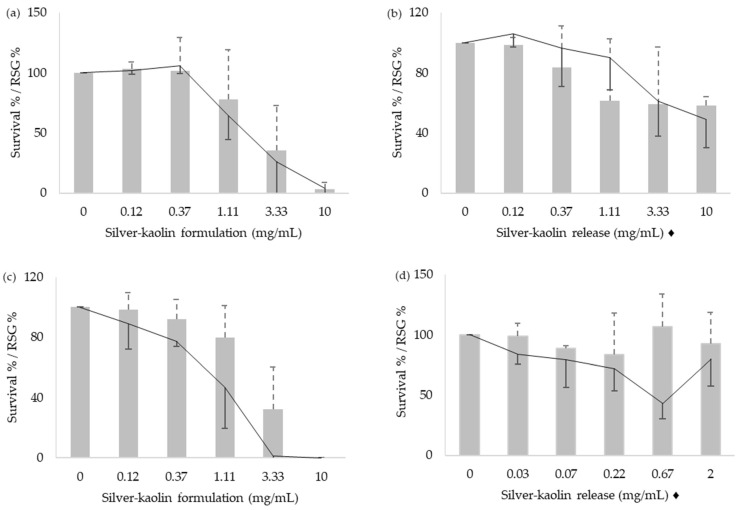
Results of the survival %, (bars with positive SD) and RSG % (lines with negative SD) of L5178Y TK^+/−^ cells. Cells were treated with the silver-kaolin formulation for 3 h (**a**), silver-kaolin release for 3 h (**b**), silver-kaolin formulation for 24 h (**c**), and silver-kaolin release for 24 h (**d**). Means and SD of three independent experiments are represented. ♦ The concentrations indicated correspond to the silver-kaolin formulation that was in agitation for 24 h (see Section 2.3).

**Figure 2:**
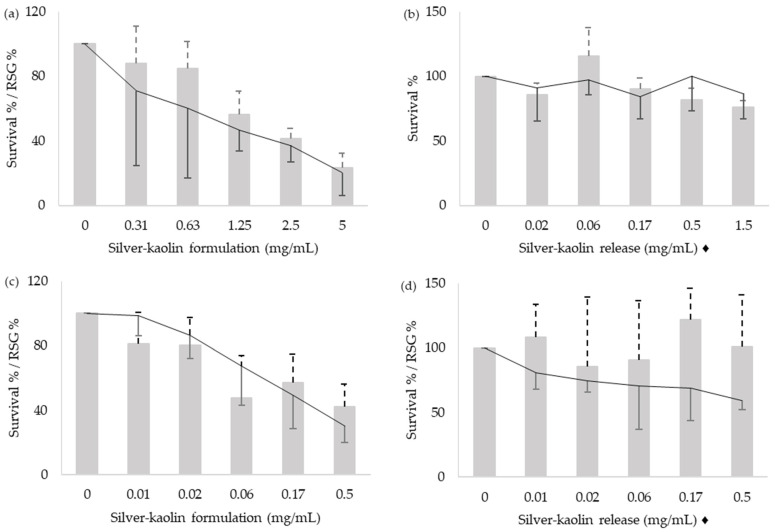
Results of the survival %, (bars with positive SD) and RSG % (lines with negative SD) of TK6 cells. Cells were treated with the silver-kaolin formulation for 3 h (**a**), silver-kaolin release for 3 h (**b**), silver-kaolin formulation for 24 h (**c**), and silver-kaolin release for 24 h (**d**). Means and SD of three independent experiments are represented. ♦ The concentrations indicated correspond to the silver-kaolin formulation that was in agitation for 24 h (see Section 2.3).

The cytotoxicity of silver-kaolin formulation was evaluated by counting the cells just after the treatment, and again 48 h after the treatment. Results of the cytotoxicity on L5178Y TK^+/−^ and TK6 cells are shown in Figure 1 and Figure 2, respectively. Both survival % and RSG % were considered as affected in values below 80%.

According to the results and the principles of the OECD TG 490, the concentrations that were chosen to be tested in the MLA were up to 3.33 mg/mL for 3 h treatment and up to 2 mg/mL for 24 h treatment. Three lower 1/3 concentrations (serial dilutions) were also tested.

For the MN test, following the principles of the OECG TG 487, the concentrations that were decided were up to 2 mg/mL for 3 h treatment and up to 0.5 mg/mL for 24 h treatment; and three lower 1/3 concentrations (serial dilutions).

Finally, for the comet assay, the concentrations tested were up to 1.5 mg/mL for the 3 h treatment and 0.5 mg/mL for the 24 h treatment; and four lower 1/3 concentrations (serial dilutions).

### 3.3. Mouse Lymphoma Assay

**Figure 3:**
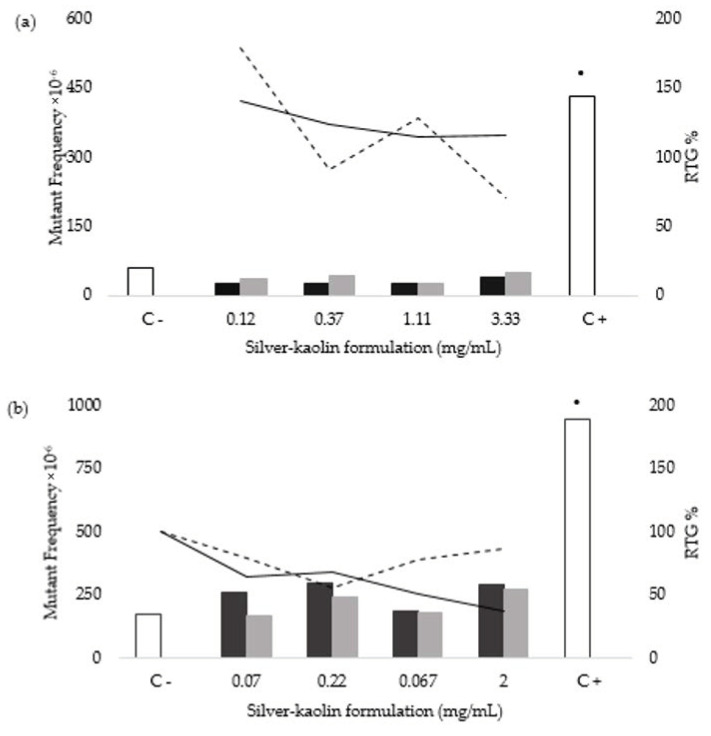
Results of the MLA after 3 h (**a**) and 24 h (**b**) treatment of L5178Y TK+/− cells. Each figure shows the induction of gene mutations, represented as mutant frequency ×10^−6^, by silver-kaolin formulation (black bars), its corresponding silver-kaolin release (grey bars), and for the negative (C−) and positive controls (C+) (white bars with black edge). It also shows the cytotoxicity of silver-kaolin formulation (continuous line) and its corresponding silver-kaolin release (dotted line), represented as % RTG values compared to the negative control for each testing condition. C+: cells treated with 100 µM MMS. **•**: difference from negative control based on OECD TG 490 global evaluation factor (GEF).

Induction of gene mutations was assessed by the MLA in L5178Y TK^+/−^ cells following the principles of the OECD TG 490 [37]. The test compound was assayed in two different forms: the silver-kaolin formulation directly added to the cell cultures, and the silver-kaolin release obtained after 24 h of continuous shaking of silver-kaolin formulation in medium. One MLA assay was carried out for each treatment time. Results are shown in Figure 3.

Neither the silver-kaolin formulation nor its corresponding silver-kaolin release produced a MF increase above the values obtained in the negative control plus GEF at any treatment conditions; thus, the material gave a clear negative response. Furthermore, concentration-related effect was not observed. Given the negative results, colony size was not considered.

### 3.4. MN Test

Induction of structural and numerical chromosome aberrations was assessed by MN test in TK6 cells following the principles of the OECD TG 487 [40]. TK6 cells were treated with silver-kaolin formulation for 24 h and with the silver-kaolin release for 3 h and 24 h. Two independent MN tests were carried out for each test compound form and treatment time. Results are shown in Table 1.

Silver-kaolin formulation produced some statistically significant MN increase above the negative control, but no testing condition reported an at least three-fold MN increase compared to the negative control. Furthermore, no significant concentration-related effect was observed through the regression test in any of the MN tests carried out.

### 3.5. Comet Assay

**Figure 4:**
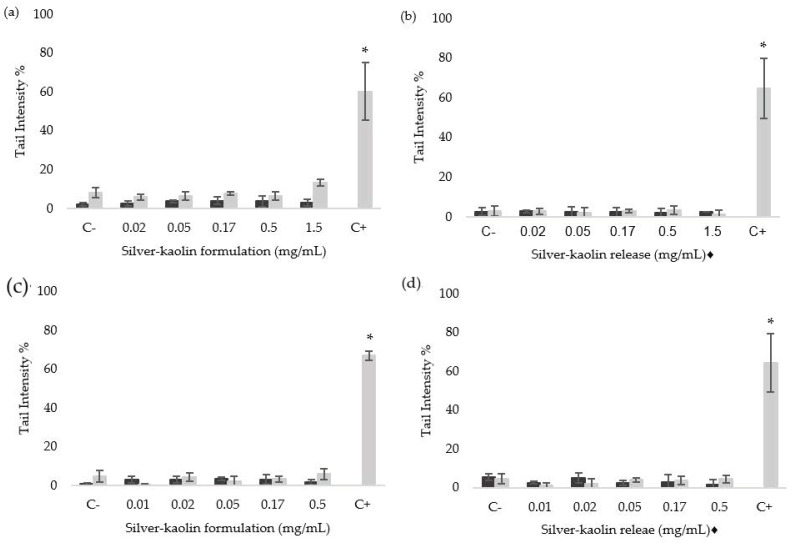
Results of comet assays after 3 h treatment with silver-kaolin formulation (**a**), 3 h treatment with the corresponding silver-kaolin release (**b**), 24 h treatment with silver-kaolin formulation (**c**), and 24 h treatment with the corresponding silver-kaolin release (**d**). Each figure shows the induction of DNA damage as SBs and ALS (black bars) and oxidized bases (Fpg-sensitive sites, grey bars) in TK6 cells, represented as % DNA in tail. Negative control (C−): untreated cells. Positive control (C+) treatment: cells treated with 1.25 mM KBrO_3_ for 3 h. The results of three independent experiments are represented in each case as mean ± SD. *: statistically significant difference from negative control (*p* < 0.05). ♦ The concentrations indicated correspond to the silver-kaolin formulation that was in agitation for 24 h (see Section 2.3).

The induction of SBs, ALS, and oxidized bases was assessed by the standard and the Fpg-modified comet assay, in TK6 cells exposed to silver-kaolin formulation and its corresponding silver-kaolin release, for 3 h and 24 h. Results are shown in Figure 4.

Neither the silver-kaolin formulation nor its corresponding silver-kaolin release produced statistically significant % DNA in tail increase for SBs, ALS, or Fpg-sensitive sites, compared to the negative control, at any treatment conditions. Furthermore, a concentration-related effect was not observed.

## 4. Discussion

Silver-kaolin formulation has been developed to be used as a feed additive in response to the growing problem posed by antimicrobial resistance, based on the known bactericidal effect of silver and the inert nature of kaolin. As an essential part of silver-kaolin formulation safety assessment, an in vitro genotoxicity evaluation has been carried out following the EFSA 2018 guideline testing strategy and the principles of their corresponding OECD guidelines. The silver-kaolin formulation and its release were evaluated through the MLA, the MN test, and the standard and Fpg-modified comet assay. The material did not induce gene mutations, chromosome aberrations, or DNA damage under the conditions tested. These results prove that the genotoxicity of AgNPs is decreased when they are conjugated with kaolin.

Characterization of the test item is an important part of the safety assessment of new formulations. The silver-kaolin formulation under study was composed by a crystal phase containing kaolinite (68%), quartz (12%), illite (13%), potassic feldspar (6%), and metallic silver (0.8%). Its structure showed laminar kaolin microparticles and spheroidal silver nanoparticles with diameters ranging from 2 to 90 nm (average diameter: 27 nm). The release of the silver contained in the formulation was assessed using SP-ICP-MS, indicating that most of the silver (Ag) released from the material in in vitro conditions was in the form of Ag+ ions, being the amount of AgNPs released very small; more than 99% were dissolved forms of Ag(I) with less than 0.1% of AgNPs (data not published).

Both the silver-kaolin formulation and its release after 24 h on cell culture medium in continuous agitation were evaluated, (see Section 2.3). Silver-kaolin formulation is a poorly soluble material and the usual centrifugation procedure to wash the cells after treatment was not useful, as the material was isolated along with the cells. Regarding cells washing setting up, several tests to separate the cells from the silver-kaolin formulation were carried out: calculations for differential centrifugation using different speeds or LymphoprepTM (i.e., lymphocytes), filtration, and decantation (data not shown). Five minutes of decantation was the selected method for the MLA, and centrifugation is selected for the MN test and the comet assays. Silver-kaolin formulation was not evaluated through the MN test after 3 h treatment, as the product was not eliminated after centrifugation and the 3 h treatment became a 24 h treatment.

According to our knowledge, there is no information available about the in vitro genotoxicity of silver-kaolin based materials. Thus, the in vitro genotoxicity evaluation of the silver-kaolin formulation was essential. The in vitro genotoxicity strategy suggested by the EFSA guideline includes assays to detect both gene mutations and chromosome aberrations [31]. The usually recommended test for the evaluation of the induction of gene mutations is the Ames test, however, it does not seem suitable for NPs assessment; instead, the MLA was carried out [31,45,46]. The induction of chromosomal aberrations was evaluated through the MN test. Both assays were performed following the principles of their corresponding OECD guidelines with slight adaptations to the material [37,40]. In this regard, it is important to emphasize the lack of protocols adapted to nanomaterials testing. The rapid growth of nanoparticles use in industry calls for a review of the current methods to correctly evaluate these materials. Moreover, a well-defined strategy for nanoparticles evaluation, composed by appropriate in vitro assays to assess a number of genotoxicity endpoints, is required to minimize extensive and costly in vivo testing [45,47,48]. Finally, the standard and Fpg-modified comet assay was also conducted to assess SBs, ALS, and DNA oxidation [31,49,50]. All assays were carried out without a metabolic activation system (S9) since poorly soluble nanomaterials are not metabolized by S9. Moreover, S9 may interfere with the assay reducing the nanomaterial bioavailability [31,51,52]. All tests were carried out under GLPs-like conditions.

The preliminary cytotoxicity of the silver kaolin formulation was performed not only to have data about the cytotoxicity of the testing compound, but more importantly, for helping in choosing the testing concentrations for the genotoxicity assays. Cytotoxicity was evaluated in preliminary assays by counting the cells, just after the treatment and 48 h after (i.e., proliferation assay). There are other methods to study the cytotoxicity of compounds (for a review check Annex I of [53]). However, since genotoxic compounds may not induce death immediately after the treatment but sometimes afterwards, the proliferation assay was the most accurate in this case. In fact, for detecting gene mutations and chromosomal aberrations, cells must undergo mitosis. Regarding the MN and the MLA assays, they also have their own cytotoxicity test that are performed at the same time as the genotoxicity assay. In the comet assay, the best measure of the cytotoxicity for a good interpretation of the results is the proliferation or the clonogenic assay [54,55,56]. In both cell lines, cytotoxicity may be caused during the treatment by a combination of physical damage produced by the collision of the suspended particles in the medium with the cells and the damage produced by the silver-kaolin release.

The highest concentration tested in MLA and MN test was established following the preliminary cytotoxicity results and the recommendations about the maximum concentration to be tested for their corresponding OECD guidelines. In the case of the in vitro comet assay, although it does not have an OECD guideline, it is the most used assay to evaluate NMs genotoxicity [57]. Therefore, it was important to establish concentrations which would show relevant results in this assay, i.e., testing non-toxic concentrations (RSG > 80%) [54,55,56]. However, an adequate number of concentrations covering a wide range of toxicity were tested in all three assays. It is worth mentioning that the cytotoxicity results obtained in the MLA and the MN test did not exactly match those of the previous proliferation assays, due to the different methods used in the preliminary cytotoxicity tests and in the main genotoxicity studies.

In the present MLA, all the acceptability criteria for the negative and positive controls were met: the negative control MF values were 60.9 × 10^−6^ and 163 × 10^−6^ for 3 h and 24 h, respectively; CE values were 71.4% and 77% for 3 h and 24 h, respectively; and TSG values were 28.1 and 78.3 3 h and 24 h, respectively. Regarding the positive controls, MF values were 431 × 10^−6^ in the 3 h treatment and 946 × 10^−6^ in the 24 h treatment, being all colonies small ones. None of the silver-kaolin formulation concentrations tested or their corresponding silver-kaolin release ones showed a positive response at any of the treatment times in comparison to their respective negative controls. Given the clearly negative results, there was no requirement for verification [37].

Regarding the MN tests, the negative and positive controls met the acceptability criteria, they were compatible with the historical control data of the toxicology laboratory, and the positive controls induced a significant increase in MN. Although in some experimental conditions the total number of MN were statistically significant in comparison to the negative control (see Section 3.4), no testing concentration induced a three-fold increase in the number of MN over the negative control. Furthermore, there was no significant concentration-related response in any case. Thus, a clear negative result was obtained. According to the OECD TG 487, a clearly negative result does not need verification [40]. However, the OECD guideline is focused on the analysis of MN on slides and not by flow cytometry. For this reason, a confirmatory experiment was performed, and similar results were obtained.

In the standard and Fpg-modified comet assays, concentrations covering a wide range of toxicity, including two toxic (RSG < 80) and three non-toxic (RSG > 80) concentrations for each treatment time, were tested. None of the concentrations tested of the silver-kaolin formulation (including the toxic ones) and their release showed any significant differences in the % DNA in tail in the case of SBs, ALS, or Fpg-sensitive sites, when compared to the negative controls.

To correctly interpret NPs negative results, it is necessary to take into account whether the material tested has been in contact with the cells or not [31,58]. In vitro internalization of different sized AgNPs in different cell lines has been widely demonstrated [59,60,61]. The direct contact of the cells with the silver released or contained in the silver-kaolin formulation was evaluated by single-cell ICP-MS. Cells containing silver were detected by this technique (Figure S3 in Supplementary Information), which implies that silver from the formulation had been internalized or adsorbed by the cells.

The silver-kaolin formulation tested in the in vitro genotoxicity assays used in this study showed clear negative results. Considering only the AgNPs content of the silver-kaolin formulation material, the concentrations tested in our assays ranged from 0.083 to 27.6 µg/mL. The concentrations of AgNPs that have been in contact with the cells are very low, which may explain the negative results. However, these concentrations are within the ranges tested in some of the studies analyzed by Rodríguez-Garraus and colleagues which did obtain positive results [32]. This may suggest that AgNPs attachment to kaolin decreases their genotoxicity, which is in accordance with other studies with similar materials [30]. It should be noted that the material was not completely removed after washing in the MLA and MN tests, it was present in all the following incubation steps, which makes the negative results even more significant. Furthermore, it is important to highlight that the silver-kaolin formulation demonstrated its in vitro antimicrobial activity at concentrations much lower than the ones tested in our studies, showing minimum inhibitory concentrations of 3.9–15.6 µg/mL and minimum bactericidal concentrations of 7.8–250 µg/mL, against several bacterial strains [25].

## 5. Conclusions

Silver-kaolin formulation seems to be a promising material to be used as an antimicrobial in animal feed. Under the conditions tested, the test compound has not produced gene mutations, chromosomal aberrations, or DNA damage (i.e., SBs, ALS, or oxidized bases) in the in vitro genotoxicity assays conducted. Yet, further in vivo genotoxicity studies should be carried out due to its complexity.

## Figures and Tables

**Table 1:** Results of the MN tests. The results of two independent experiments are represented in each case as MN/1000 Nuclei and RS %. Statistically significant difference from negative control *: (*p* < 0.05), **: (*p* < 0.01).

	Test 1		Test 2	
Silver-kaolin release ♦ (mg/mL) 3 h treatment	MN/1000 Nuclei	RS %	MN/1000 Nuclei	RS %
0	1.18	100	0.49	100
0.07	0.94	112	0.29	87
0.22	1.15	93	0.79	97
0.67	0.93	103	0.63	89
2	1.64	128	0.75	67
MMS 150 µM	16.77 **	38	7.53 **	47
Silver-kaolin formulation (mg/mL) 24 h treatment	MN/1000 Nuclei	RS %	MN/1000 Nuclei	RS %
0	1.71	100	3.40	100
0.02	3.65 *	85	4.60	177
0.05	4.07 *	93	3.21	109
0.17	3.21*	90	7.35 **	133
0.5	1.30	85	7.21 **	65
Colchicine 10 ng/mL	55.25 **	38	78.40 **	74
Silver-kaolin release ♦ (mg/mL) 24 h treatment	MN/1000 Nuclei	RS %	MN/1000 Nuclei	RS %
0	3.74	100	1.73	100
0.02	3.34	102	4.36 **	89
0.05	5.52 **	93	1.27	75
0.17	3.44	81	1.32	77
0.5	2.64	93	2.25	73
Colchicine 10 ng/mL	42.28 **	59	95.90 **	23

♦ The concentrations indicated correspond to the silver-kaolin formulation that was in agitation for 24 h (see Section 2.3).

## Data Availability

Not applicable.

## Supplementary Materials

The following supporting information can be downloaded at: https://www.mdpi.com/article/10.3390/nano12060914/s1. Figure S1: FESEM images of the silver-kaolin formulation. Figure S2: Weight size distribution of the silver-kaolin formulation. Figure S3: Mass distribution of silver internalized by L5178Y TK^+/−^ cells obtained by SC-ICP-MS analysis. Short treatment (10 mg/mL).
