# Supplementary material for: In Vitro Genotoxicity Evaluation of an Antiseptic Formulation Containing Kaolin and Silver Nanoparticles
Source: Nanomaterials (Basel). 2022 Mar 10;12(6):914. doi: 10.3390/nano12060914 (PMC8948953; doi:10.3390/nano12060914)
Supplement: Supplementary file 1 [file nanomaterials-12-00914-s001.zip › nanomaterials-1551476-supplementary.pdf]

SUPPLEMENTARY INFORMATION

Characterization of the silver-kaolin formulation

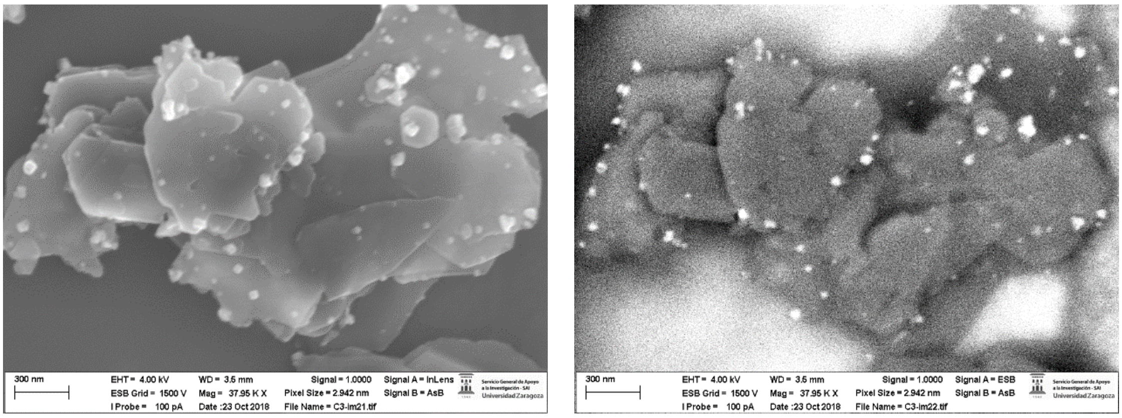

Figure S1. FESEM images of the silver-kaolin formulation.

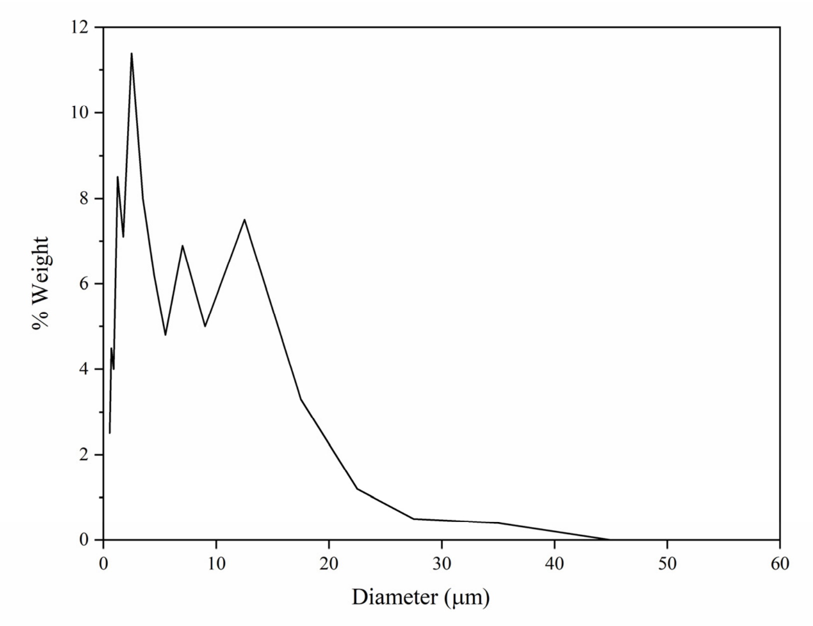

Figure S2. Weight size distribution of the silver-kaolin formulation.

Single cell ICP-MS

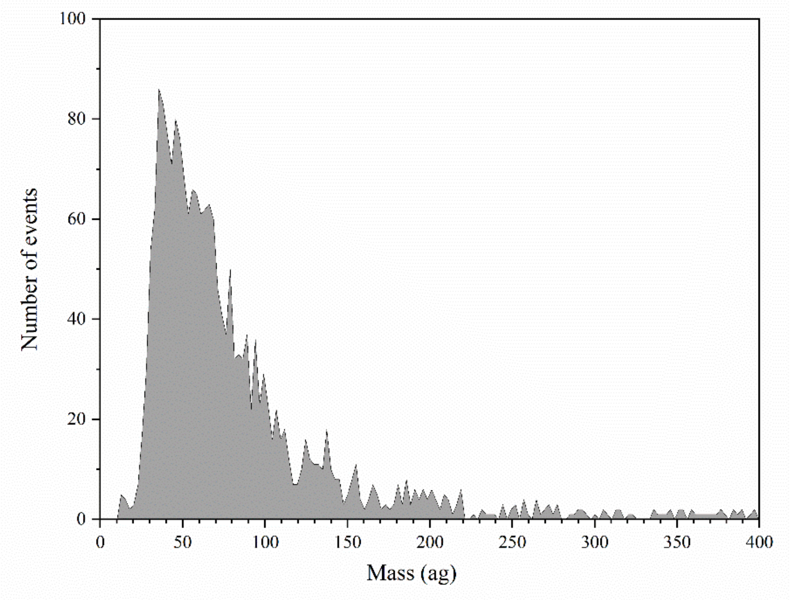

Figure S3. Mass distribution of silver internalized by L5178Y TK$^{+/-}$ cells obtained by SC-ICP-MS analysis. Short treatment (10 mg/mL).

Cell suspension obtained from the exposure experiments was washed three times with ML10 and diluted with PBS to get a concentration in number of cells of approximately 10^4-10^5 cells/mL. Single-cell analysis was performed by using a Perkin-Elmer NexION 2000 mass spectrometer (Toronto, Canada). The sample introduction system consisted of a high efficiency nebulizer and an Asperon spray chamber. Suspensions were measured in single cell mode using the Syngistix Nano-Application module version 2.5 (PerkinElmer Inc.) The dwell time used was 100 s with a total acquisition time of 60 s, and samples were introduced into the plasma at a flow rate of 15 L/min.

Mass distribution obtained by single cell inductively coupled plasma mass spectrometry (SC-ICP-MS) analysis showed the silver content (internalized or adsorbed by the cells) corresponding to individual cells, in units of attograms/cell. The technique cannot distinguish between dissolved silver and silver nanoparticles inside the cells.
